# Coupling Seq-BSA and RNA-Seq Analyses Reveal the Molecular Pathway and Genes Associated with Heading Type in Chinese Cabbage

**DOI:** 10.3389/fgene.2017.00176

**Published:** 2017-12-12

**Authors:** AiXia Gu, Chuan Meng, YueQi Chen, Lai Wei, Hui Dong, Yin Lu, YanHua Wang, XuePing Chen, JianJun Zhao, ShuXing Shen

**Affiliations:** ^1^Key Laboratory of Vegetable Germplasm Innovation and Utilization of Hebei, Collaborative Innovation Center of Vegetable Industry in Hebei, College of Horticulture, Hebei Agricultural University, Baoding, China; ^2^Economic Crop Research Institute, Hebei Academy of Agriculture and Forestry Sciences, Shijiazhuang, China; ^3^Shijiazhuang Pomology Institute, Hebei Academy of Agriculture and Forestry Sciences, Shijiazhuang, China

**Keywords:** Chinese cabbage, heading type, plant hormone, *BrGH3.12*, *BrABF1*, Seq-BSA, RNA-Seq

## Abstract

In Chinese cabbage, heading type is a key agricultural trait of significant economic importance. Using a natural microspore-derived doubled haploid plant, we generated self-crossed progeny with overlapping or outward curling head morphotypes. Sequencing-based bulked segregant analysis (Seq-BSA) revealed a candidate region of 0.52 Mb (A06: 1,824,886~2,347,097 bp) containing genes enriched for plant hormone signal transduction. RNA Sequencing (RNA-Seq) analysis supported the hormone pathway enrichment leading to the identification of two key candidate genes, *BrGH3.12* and *BrABF1*. The regulated homologous genes and the relationship between genes in this pathway were also revealed. Expression of *BrGH3.12* varied significantly in the apical portion of the leaf, consistent with the morphological differences between overlapping and outward curling leaves. Transcript levels of *BrABF1* in the top, middle and basal segments of the leaf were significantly different between the two types. The two morphotypes contained different concentrations of IAA in the apical portion of their leaves while levels of ABA differed significantly between plant types in the top, middle, and basal leaf segments. Results from Seq-BSA, RNA-Seq and metabolite analyses all support a role for IAA and ABA in heading type formation. These findings increase our understanding of the molecular basis for pattern formation of the leafy head in Chinese cabbage and will contribute to future work developing more desirable leafy head patterns.

## Introduction

Chinese cabbage (*Brassica rapa* ssp. *pekinensis*) is widely cultivated in Asia and is becoming increasingly more popular in other countries. As a leafy vegetable crop, leaves at the seedling and rosette stage function primarily in photosynthesis and respiration. At the heading stage, a tight, leafy head is formed and serves as a storage organ and an edible product. Chinese cabbage heading type is an important commercial trait indicating how the top of leaves form the head. The patterns include overlapping, outward-curling, inward-curling without overlap, and spiral (Xu et al., 2004). The most commonly cultivated is the overlapping type, where the heading leaves curl inward at the top with the curling length exceeding the vertical central axis of the leafy head. This is the preferred type by both growers and consumers because it presents a closed top, cleaner inner leaves, a higher net-to-head ratio, and facilitates easier packaging and transportation. In contrast, outward-curling types produce leaves that curl outward and appear open at the top. The molecular regulatory mechanism responsible for heading type in Chinese cabbage remains elusive and has not to our knowledge been previously described.

The molecular mechanism of leafy head formation is also unclear (Wang et al., 2014), although some progress has been made. Changes in concentration of the plant hormone auxin (indole-3-acetic acid, or IAA) between the adaxial and abaxial sides were shown to cause leaves to curl inward to form a leafy head (Li, 1984). He et al. (2000) introduced IAA-related genes into Chinese cabbage and found that the transgenic plants showed an earlier heading date, produced more leaves, and developed heavier heads. Other studies have focused on head weight, diameter, and height, as well as heading time (Yu et al., 2013; Inoue et al., 2015); or the genes responsible for leaf curvature (Xiao et al., 2014). Based on results from adaxial-abaxial (ad-ab) polarity research in *Arabidopsis thaliana* (Fukushima and Hasebe, 2014), *Brassica rapa* ssp. *pekinensis TEOSINTE BRANCHED1, cycloidea, and PCF transcription factor 4* (*BrpTCP4*) was shown to affect the leafy head size and shape of Chinese cabbage (Mao et al., 2014) while *BrpSPL9* (*B. rapa* ssp. *pekinensis SQUAMOSA PROMOTER BINDING-LIKE 9-2*) was found to influence the timing of leafy head formation (Wang et al., 2014). The roles of *BrARF3.1 (AUXIN RESPONSE FACTOR 3)* and *BrKAN2.1 (KANADA2)* in determining the formation of leafy heads has also been examined through comparative genomic analysis (Cheng et al., 2016; Liang et al., 2016), while Genome-Wide Association Studies (GWAS) have been used to investigate the effect of *WRKY* and *F-box* genes on head diameter (Lim et al., 2015). Finally, through RNA-seq techniques, genes that are differentially expressed between the rosette and heading stages have been classified into four groups: transcription factors, protein kinases, calcium signaling, and auxin-related genes (Wang et al., 2012).

**Figure 1:**
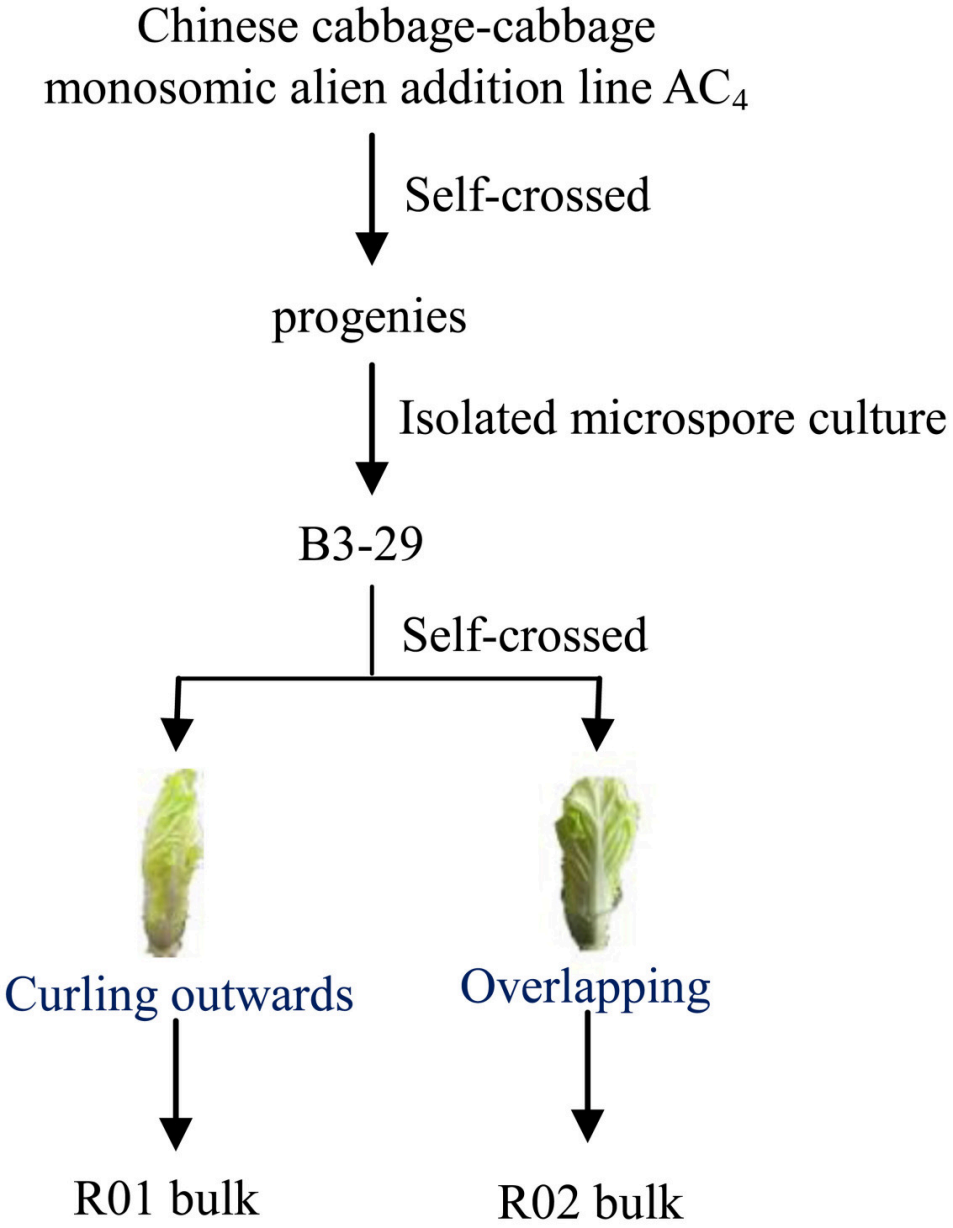
Schematic representation of origins for experimental materials.

In this study, we obtained a natural microspore-derived doubled haploid plant, B3-29, from an introgression line containing chromosome segments of cabbage (*B. oleracea* var. *capitata*) in a Chinese cabbage background (Qin, 2013). Although the morphological phenotypes of individual field-grown plants were nearly identical during the seedling and rosette stages, two phenotypes of heading type were observed during the heading stage, overlapping and outward-curling (Figure 1; Dong, 2015). This provided a new opportunity for studying the heading type phenomenon. The availability of the Chinese cabbage genome (Wang, X. et al., 2011) facilitates the investigation of the molecular mechanisms responsible for the heading type trait. To identify the pathways and genes related to these two heading type phenotypes we applied a Sequencing-based Bulked Segregant Analysis (Seq-BSA) combined with RNA-Seq. The results were further validated at the metabolite level. Our findings provide the first molecular evidence of heading type formation using whole genome analysis in Chinese cabbage.

## Materials and methods

### Plant materials

The natural microspore-derived doubled haploid plant B3-29 used in this experiment was obtained by isolating microspore culture from M50-12 (Qin, 2013), which was derived from self-crossed progenies of a *Brassica rapa*-*Brassica oleracea* monosomic alien addition line AC_4_ (Gu et al., 2015). After nine generations of subculturing, seeds of B3-29 were planted in the field in January of 2013. In August of 2014, 125 seeds of selfed B3-29 were sown. The surviving plants displayed two contrasting heading type phenotypes: overlapping (82 plants) and outward-curling (31 plants) (Figure 1). From these, we selected 25 plants of each morphotype and used them to establish R01 and R02 bulks for Seq-BSA analysis. RNA-Seq was performed on three plants from each bulk.

### Methods

#### Trait observations

Leaf growth rates were monitored from the rosette stage to the early heading stage of Chinese cabbage using 10 plants each of the overlapping and outward-curling types. The maximum length and width of leaves was recorded in 2-day intervals to track leaf growth. At the heading stage (65 days after transplanting), we examined the maximum width and length of leaves as they were naturally displayed as well as when individual leaves were spread out and pressed until almost completely flat. Total weight and the number of leaves were determined from three plants for each morphotype. For vasculature observations, the largest head-forming leaves were cleared of chlorophyll in 95% alcohol overnight and treated with distilled, deionized water: glycerol: phenol: lactic acid (1:1:1:1) for 20 min at 90°C.

#### Seq-BSA analysis

##### Illumina library construction and sequencing

A total of 50 plants (25 overlapping, 25 outward-curling) were used for sequencing. Genomic DNA was isolated from young leaves with an extraction kit (Aidlab Biotechnologies Co. Ltd., China), and two DNA pools from R01 and R02 bulks were prepared by mixing equimolar concentrations of DNA samples from each morphotype. The Illumina libraries for these two pools were prepared using a NEBNext® DNA Library Prep Reagent Set for Illumina® (E6000L; Illumina Inc., San Diego, CA, USA). A total of 5 μg of DNA from each sample was sheared via Ultrasonic cleaning (KQ-50E; Kunshan Ultrasonic Instrument Co. Ltd.,) followed by end-repair and adapter-ligation. Size selection of libraries was performed using a 2% agarose gel to obtain a target insert size of 500 bp. The purified libraries were amplified using adaptor-compatible PCR primers and the size distribution was assessed on an Agilent 2100 Bioanalyzer (Agilent Technologies, Palo Alto, CA, USA). The DNA libraries were sequenced on an Illumina HiSeq 2500 platform with an FC-401-4002_HiSeq SBS Kit V4 (50 cycles) and a PE-401-4001_HiSeq PE Cluster Kit V4 cBot (Illumina Inc.,) to generate 125 bp paired-end reads.

##### Sequence alignment and variant calling

A custom-built program in C++ was used to filter out low-quality reads and duplicates were discarded using SAMtools (Li et al., 2009). Filtered reads were aligned to the reference Chinese cabbage genome (http://brassicadb.org/brad/datasets/pub/Genomes/Brassica_rapa/V1.5/) using BWA (Li and Durbin, 2009). The GATK Toolkit (McKenna et al., 2010) was used to correct for alignment errors due to local rearrangements around insertions and deletions as well as recalibration of read-base quality and variant calling. The variants were removed if the distance was less than 10 bp between two indels, if more than two SNPs occurred in a 5 bp window, or SNPs were within 5 bp of an indel.

##### Mapping of candidate genomic regions

Candidate regions were selected based on Euclidean distance (ED) associations (Hill et al., 2013). After the loci for multiple mutations, undetected sites in any single bulk, or homozygous and consistent loci in both bulks were filtered out, the ED was calculated at each SNP location using the following equation:


$$ED=\sqrt{{\left(A_{R01}-A_{R02}\right)}^{2}+{\left(C_{R01}-C_{R02}\right)}^{2}+{\left(G_{R01}-G_{R02}\right)}^{2}+{\left(T_{R01}-T_{R02}\right)}^{2}}$$

Where A, C, G, and T represented their corresponding bases. The ED was then raised to a power (default = 5), without masking repetitive regions (default = not masked). These data were subsequently passed to R for signal processing and peak identification. The ED values across the genome were fitted and sliding-window averages of 1 Mb were plotted. Peak regions were defined as candidate regions where the Distance-fitted values were greater than three standard deviations above the genome-wide median. From this, R could be used to plot Distance fits.

#### RNA-Seq analysis

At the early heading stage (68 days after sowing), we sampled the ninth leaf in from the exterior of the developing head at three distinct positions: apical (S), middle (Z, corresponding to the bend position on the overlapping morphotype), and basal (X) (Figure S2). The morphotypes were referred to as DB for overlapping and SX for outward-curling. Each sample was divided into two sets: one for RNA-Seq, the other for determining IAA and ABA concentrations.

##### RNA extraction and quality test

Total RNA was isolated with an RNA extraction kit (Huayueyang, China), and quantified with a Qubit® RNA Assay Kit on the Qubit® 2.0 Fluorometer (Life Technologies, CA, USA). The RNA integrity was assessed on the Bioanalyzer 2100 System using the RNA Nano 6000 Assay Kit (Agilent).

##### RNA-Seq library construction and sequencing

A total of 3 μg RNA per sample was used as input material for the RNA sample preparations. Our RNA-Seq libraries were constructed with a NEBNext® Ultra™ RNA Library Prep Kit for Illumina® (NEB, USA). Briefly, mRNA was purified from total RNA using poly-T oligo-attached magnetic beads. Fragmentation was performed using divalent cations at an elevated temperature in the NEB proprietary fragmentation buffer (5X). The RNA-Seq libraries were sequenced on an Illumina HiSeq 4000 platform to generate 150 bp paired-end reads.

##### Bioinformatics analysis of RNA-Seq data

Raw reads were pre-processed to remove adapter, poly-N sequences and low quality reads. Processed reads were mapped to the Chinese cabbage reference genome using TopHat (Trapnell et al., 2009, 2012) v. 2.0.12. Read counts for each gene were summarized by HTSeq (Anders, 2010) v. 0.6.1. and FPKM values were calculated based gene length and read counts mapping to the gene. Differential expression analysis of the two groups (three biological replicates per group) was performed using the DESeq package (Anders and Huber, 2010, 2012; Wang et al., 2010) in R (1.18.0). This software provides statistical routines for determining differential expression using a negative binomial distribution model. The resulting *P*-values were adjusted using the Benjamini and Hochberg method to control for false discovery rates. Genes with an adjusted *P* < 0.05 were considered differentially expressed.

##### Real-time quantitative RT-PCR

The qRT-PCR verifications were conducted as previously described (Gu et al., 2016), using a Thunderbird SYBR qPCR Mix (Toyobo, Shanghai, China) and a LightCycler® 96 (Roche). All primers are listed in Table S1.

#### Quantification of IAA and ABA

Concentrations of IAA and ABA were determined using a liquid chromatography–tandem mass spectrometry (LC–MS/MS) system [MS API 4000 (Applied Biosystems, USA) and LC 1200 (Agilent)]. Leaf samples were frozen in liquid nitrogen and 200-mg portions were ground, placed in ultrasonic pots and extracted in 3 mL of solution (n-propyl alcohol/water/hydrochloric acid; v/v/v = 200.0/100.0/0.2) for 30 min in an ice bath in the dark. Following incubation, 2 mL of dichloromethane was added to the extraction and incubated for 30 min. The substratum liquid was collected following centrifugation at 4,500 rpm for 10 min, dried under nitrogen and re-dissolved in 1 mL of 80% (v/v) methanol. The supernatants were collected after centrifugation at 12,000 rpm for 10 min and passed through a 0.22-μm filter membrane. After 200 μL of this supernatant was loaded onto the LC-MS/MS system, the procedure continued on a 2.1 mm × 50 mm XBridgeTM C18 2.5 μm column (Waters, USA), at a column temperature of 30°C. The mobile phase comprised solvent A (0.05% HCOOH, v/v) (Waters) and solvent B (0.05% HCOOH-CH_3_CN, v/v) in a gradient mode (time/concentration of A/concentration of B) of 0.01/0/10, 2.0/90/10, 12.0/50/50, 12.1/90/10, and 15/90/10, at a flow rate of 0.2 mL min^−1^. The MS settings were as follows: capillary voltage, −4.5 kV; source temperature, 350°C; scan type, multiple reaction monitoring (MRM); dwell time, 200 ms; settling time, 700 ms; and MR pause, 5.007 ms. For IAA, the MRM was set at: Q1/Q3 174/130, CE −13, DP −43, CXP −21, and EP −10; whereas for ABA, the MRM was set at: Q1/Q3 137/93, CE −22, DP −37, CXP −16, and EP −10.

#### Statistical analysis

Significant differences in phenotypic traits and hormone levels between overlapping and outward-curling plants was assessed by Fisher's exact test using a *P* < 0.05.

Genes in the candidate region from the Seq-BSA analysis were mapped to the Kyoto Encyclopedia of Genes and Genomes (KEGG) database (http://www.genome.ad.jp/kegg/; Kanehisa et al., 2004) to classify genes based on biological function. Pathway enrichment for genes in the candidate region was determined using the entire genome as the background set. Statistical significance was assessed by Fisher's exact test using a *P*<0.05.

We used KOBAS software (Mao et al., 2005) to test the statistical enrichment of differentially expressed genes in KEGG pathways.

## Results

### Characteristics of overlapping and outward-curling morphotypes

**Figure 2:**
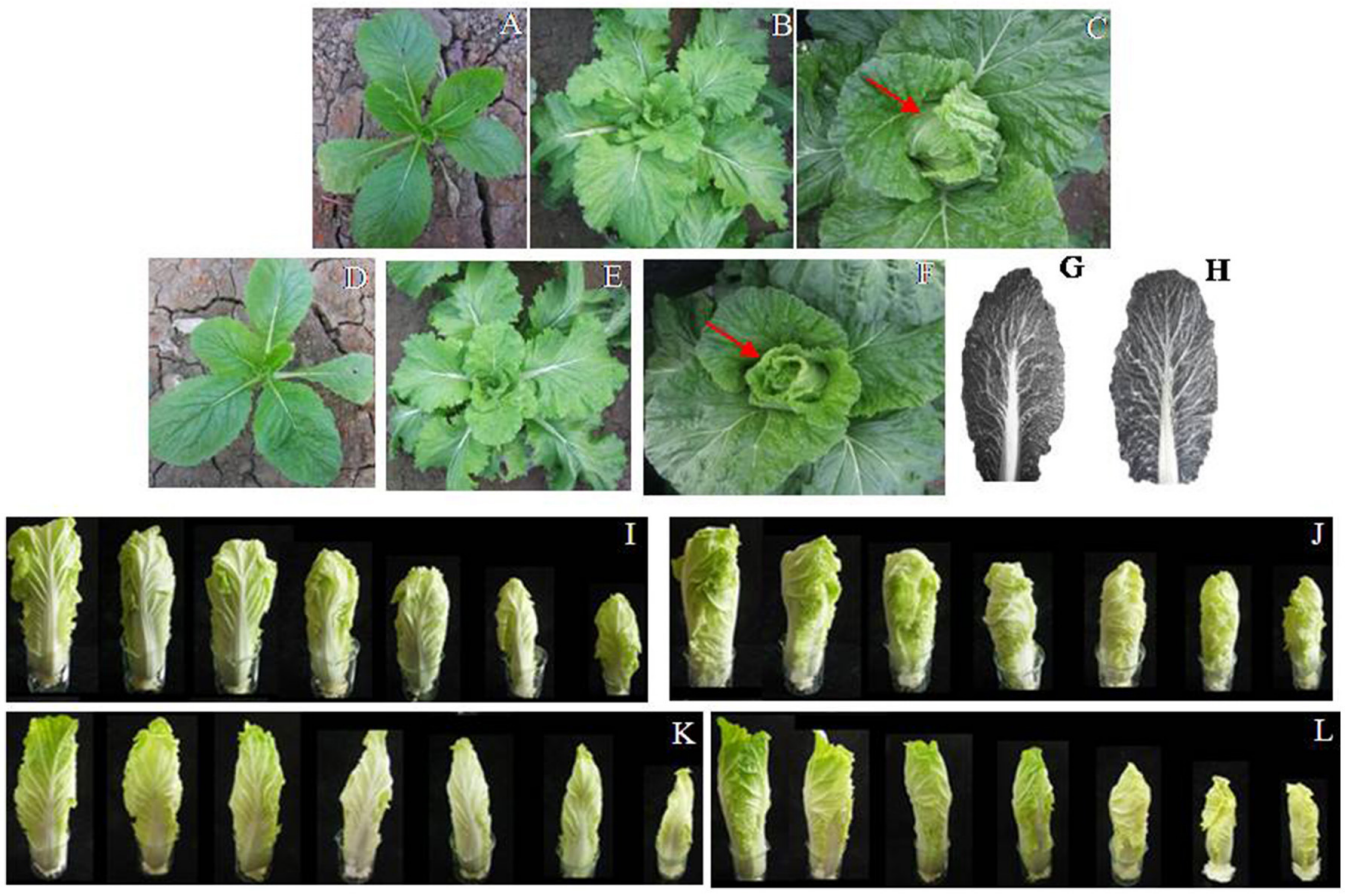
Two types of head leaf patterns in top region during the formation of heads (red arrows) for self-crossed progenies from line B3-29. Overlapping type: **(A)** seedling stage; **(B)** rosette stage; **(C)** heading stage. Outward-curling type: **(D)** seedling stage; **(E)** rosette stage; **(F)** heading stage. Leaf veins: **(G)** overlapping type; **(H)** outward-curling type. Appearance after head-forming leaves were removed in individual layers (left to right, outer to innermost layer): **(I,J)**, overlapping type; **(K,L)**, outward-curling type.

From seedling to the early heading stage, we did not observe noticeable differences in phenotype (Figures 2A,B,D,E) or leaf growth over time (Figure S1). However, their respective heading type showed visible distinctions during head formation between overlapping (Figures 2C,I,J) vs. outward-curling types (Figures 2F,K,L). When measurements were taken of heads in their natural state, leaves were longer in the outward-curling group, and differences were not significant (*P* = 0.28). However, when completely spread out, the leaves were significantly longer in the overlapping group (*P* = 0.02). In both measurements, the leaf width was similar between types (*P* = 0.52, natural state; *P* = 0.78, completely spread out). On average, the outward-curling plants were slightly heavier (*P* = 0.55) and produced significantly more leaves than the overlapping type (*P* = 0.01). The overlapping plants showed more branching of the leaf veins, with at least three secondary veins produced for each primary vein resulting in a greater number of small secondary veins (Figure 2G). By comparison, the primary veins on the outward-curling leaves were spread more widely toward the leaf edge with barely visible secondary veins (Figure 2H).

### Seq-BSA analysis

#### Construction and sequencing of R01 and R02 bulks

Two Illumina libraries (R01 and R02) were constructed and subjected to whole-genome re-sequencing using Illumina HiSeq2500. In total, 123.18 million paired-end (PE) reads (60.88 million and 62.31 million reads for R01 and R02, respectively) were generated. Mapping of those reads to the reference genome resulted in 23X and 28X coverage with 87.40 and 86.31% mapping efficiency for R01 and R02 bulks, respectively (Table 1).

**Table 1:** Sequencing and mapping of sequence reads.

**Bulk**	**Clean reads**	**Data generated (Gb)**	**Q30 (%)**	**Genome coverage (%)**	**Average depth (X)**	**SNP number**
R01	121763612	15.34	85.73	87.40	23	1328929
R02	124615198	15.70	86.86	86.31	28	1336057

#### Selection of candidate regions

**Figure 3:**
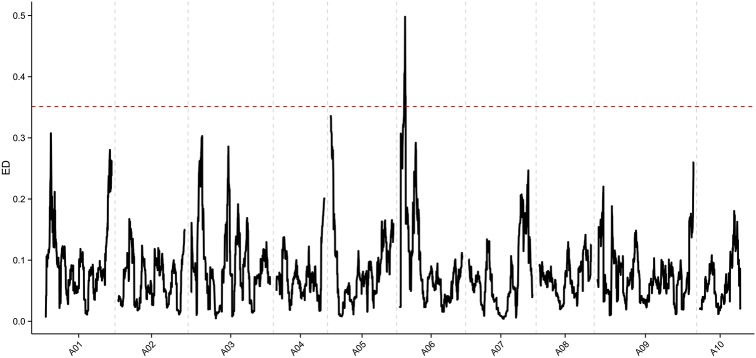
Values calculated for ED on chromosomes, based on Seq-BSA analysis.

We used 72,962 SNPs between R01 and R02 for association analysis. Values for Euclidean distance (ED) were fit across the genome and sliding-window averages of 1 Mb were plotted. The ED threshold was 0.35 when three standard deviations above the genome-wide median were chosen. The candidate regions responsible for heading type in overlapping and outward-curling types were selected from peaks with an ED above the threshold. From this, one candidate region at chromosome A06 was detected, with a total length of 0.52 Mb (1,824,886 ~ 2,347,097 bp) (Figure 3) containing 90 genes (Table S2).

#### Analysis of genes in the candidate region

To identify the biological pathways that are active in the formation of heading type, we mapped 90 candidate genes to KEGG database and compared their enrichment against the whole genome. The proportion of genes that were enriched for plant hormone signal transduction (ko04075) was significant (*P* = 0.02) and contained the greatest representation by unique genes. Of these, four were enriched for plant hormone signal transduction including eight parallel pathways (auxin, cytokinin, gibberellins, ABA, ethylene, brassinosteroid, jasmonic acid, and salicylic acid), with three (Bra018749, Bra018750, and Bra018751) assigned to the auxin pathway and one (Bra018800) to the ABA pathway. One of the genes in the auxin pathway was *BrGH3.12*, an ortholog of the early-auxin-response gene *GRETCHEN HAGEN3* (*GH3*) (Hagen and Guilfoyle, 2002; Staswick et al., 2005; Bitto et al., 2009; Westfall et al., 2010, 2012; Peat et al., 2012). In the ABA pathway, we identified *BrABF1* (Bra018800), an ortholog of *ABSCISIC ACID RESPONSIVE ELEMENT BINDING FACTOR1* (*ABF1*) (Yoshida et al., 2010, 2015).

Swissport annotation and results from our literature search (Table 2) revealed six additional auxin-related genes involved in auxin biosynthesis, transport, and binding proteins, as well as an additional gene involved in the ABA response. Gene Ontology enrichment also identified the four genes mentioned above for the hormone signal transduction pathway, two (Bra018787 and Bra018772) for the auxin response, and two (Bra018787 and Bra018745) for the ABA response. The consensus results between the two database enrichment tests suggests that genes related to auxin and ABA pathways are important candidates for heading type formation in Chinese cabbage.

**Table 2:** Functions of auxin- and ABA-related genes, based on Swissport annotation.

**Hormone**	**Function**	**Gene name**	**Gene ID**
Auxin	Biosynthesis	*YUCCA* (Mashiguchi et al., 2011; Won et al., 2011; Zhao, 2012)	Bra018763, Bra018766, Bra018761
	Receptor system	*Arabidopsis SKP1-like 1* (*ASK1*) Grones and Friml, 2015	Bra018756, Bra018757
	Transporter	*WALLS ARE THIN1 (WAT1*) (Ranocha et al., 2013)	Bra018748
ABA	Response	*RESPONSIVE TO DEHYDRATION 22* (*RD22*) (Zhao, Y. et al., 2013; Harshavardhan et al., 2014)	Bra018785

### RNA-Seq analysis

#### Comparison of gene expression between morphotypes

**Figure 4:**
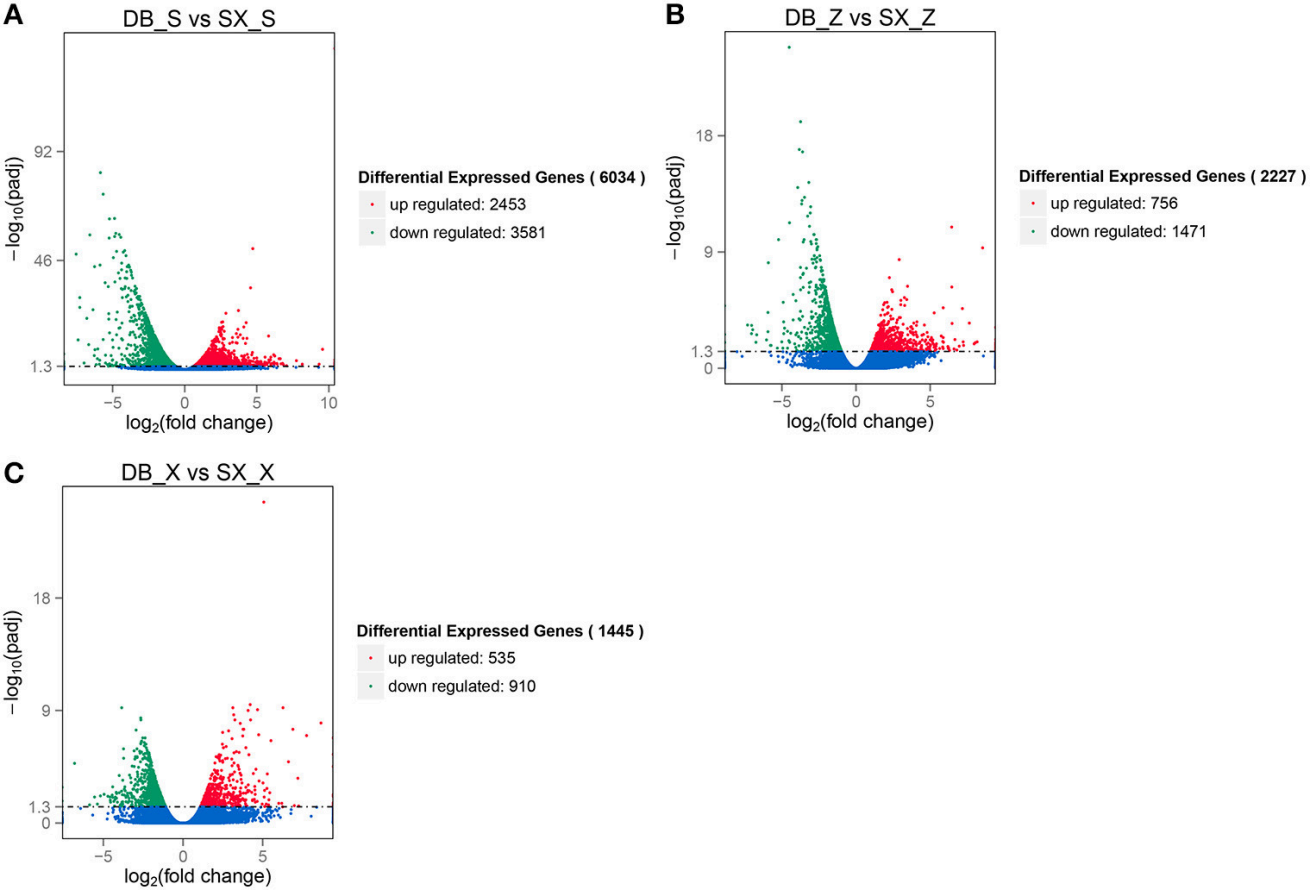
Volcano plots of DEGs between overlapping and outward-curling types. **(A)** DB_S vs. SX_S; **(B)** DB_Z vs. SX_Z; **(C)** DB_S vs. SX_S.

The RNA-Seq analysis generated 42.27–55.95 million reads for each sample. Three biological replicates were performed for each line. After removal of adaptor sequences, duplicated sequences, ambiguous reads, and low-quality reads, 30.60–41.11 million reads were mapped to the *Brassica rapa* genome (Table 3). Expression was normalized and differentially expressed genes (DEGs) were detected between the morphotypes. The following codes were used to describe the formation patterns and locations along the leaf where samples were taken: DB (overlapping) vs. SX (outward-curling); S, Z, and X to represent the top, middle, and basal portion of the leaf, respectively. From this analysis, we found 6034 DEGs between DB_S and SX_S, with 2453 genes significantly up-regulated and 3581 significantly down-regulated (Figure 4A). Among the 2227 DEGs between DB_Z and SX_Z, 756 were significantly up-regulated and 1471 were significantly down-regulated (Figure 4B). Finally, 1445 DEGs were detected between DB_X and SX_X, with 535 significantly up-regulated and 910 significantly down-regulated (Figure 4C). Thus, with regard to leaf position, when morphological differences were more significant, more DEGs were detected.

**Table 3:** Summary of transcriptome sequencing data.

**Sample**	**Clean reads**	**Q30 (%)**	**Total mapped**	**Uniquely mapped**
DB_S	43377979	96.85	31981991 73.73%	31402433 72.39%
DB_Z	55948586	97.00	41112704 73.48%	40214398 71.84%
DB_X	44958501	96.85	32878413 73.14%	32123772 71.45%
SX_S	42791156	96.52	31444702 73.49%	30836394 72.07%
SX_Z	48400741	95.78	34768856 71.88%	34123332 70.54%
SX_X	42270630	96.78	30598448 72.45%	30002739 71.04%

#### Analysis of the biological pathways involved in heading type formation in Chinese cabbage

**Figure 5:**
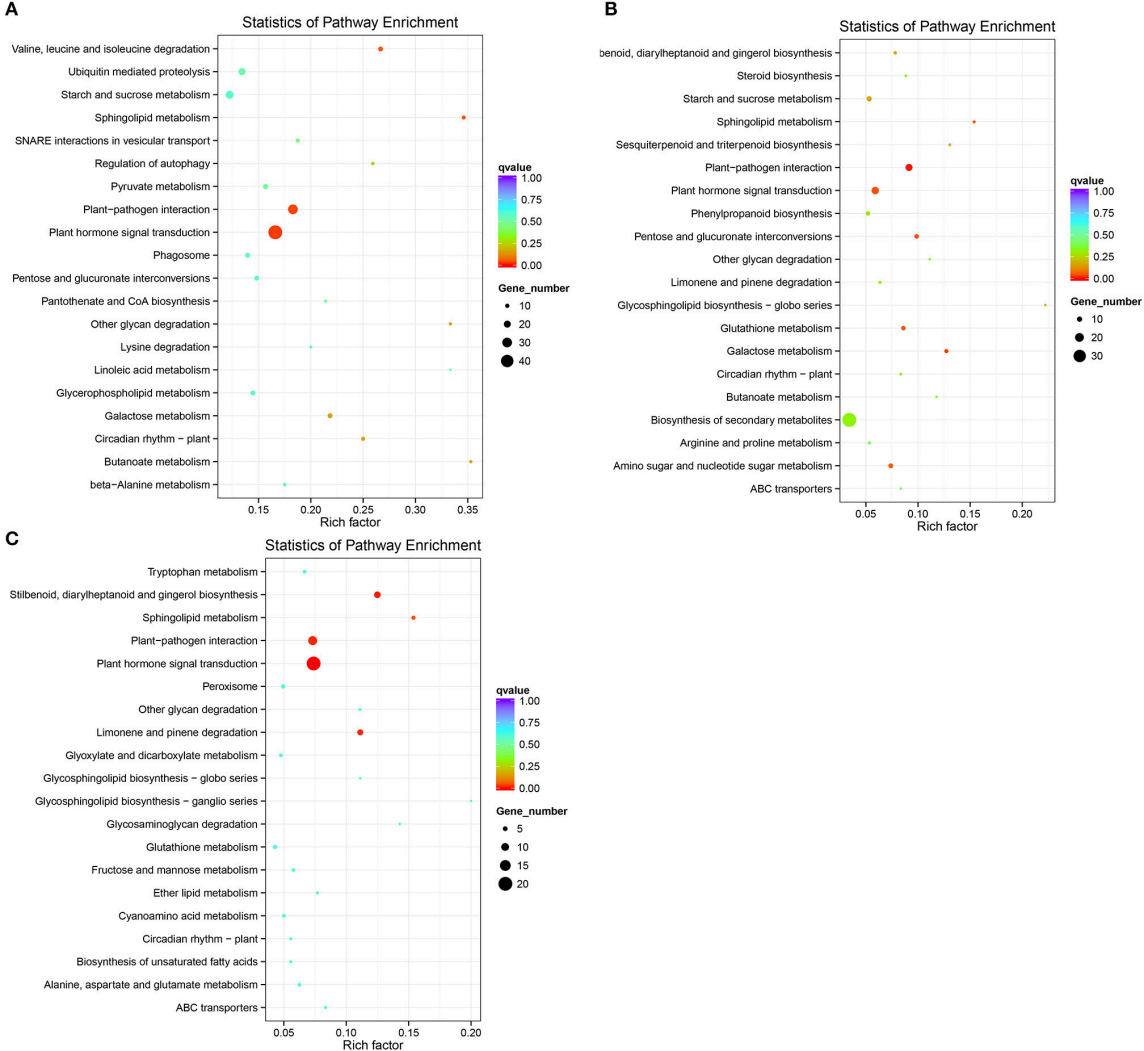
Enriched KEGG pathway scatterplots for upregulated DEGs. **(A)** DB_S vs. SX_S; **(B)** DB_Z vs. SX_Z; **(C)** DB_S vs. SX_S.

We mapped the DEGs to the reference canonical pathways in KEGG. Up-regulated DEGs with roles in plant hormone signal transduction were significantly enriched in the three leaf positions between each heading type: DB_S vs. Sx_S, DB_Z vs. Sx_Z and DB_X vs. Sx_X (Figure 5). The corrected *p*-values (*q*-values) indicated that these enrichments were statistically significant at 0.02, 0.04, and 0.00, respectively. Furthermore, the candidate genes overlapped with the genes identified from our Seq-BSA analysis, providing additional support for the role of plant hormone signal transduction in specifying heading types in Chinese cabbage. Among the eight parallel pathways, the most significantly enriched were auxin and ABA (Table S3). The proportions of up-regulated DEGs in the auxin and ABA pathways compared to the overall pathway were 17.78 and 24.44%, respectively, when DB_S was compared with SX_S. For DB_Z vs. SX_Z, the proportions were 43.75 and 31.25%, respectively; while those proportions were 30.00 and 55.00% for DB_X vs. SX_X. Based on the Seq-BSA analysis, genes in the candidate region related to plant hormone signal transduction were entirely committed to the auxin and ABA pathways. Therefore, the results from the Seq-BSA and RNA-Seq analysis are consistent.

#### Identification of candidate genes

Among the candidates related to auxin and ABA signaling detected in the Seq-BSA analysis, two (*BrGH3.12* and *BrABF1*) exhibited significantly different expression in the RNA-Seq data. Transcript levels of *BrGH3.12* and *BrABF1* were significantly different between types when measured at the apical region of the leaf. Furthermore, *BrABF1* expression was significantly different between types when detected at the middle and basal positions. Therefore, the combination of Seq-BSA and RNA-Seq analyses indicated that *BrGH3.12* and *BrABF1* are strong candidate genes for heading type.

#### Expression changes of auxin and ABA-related genes

The DEGs assigned to the auxin pathway (Table S4) when DB_S was compared to Sx_S showed that one orthologs of *TIR1* (TRANSPORT INHIBITOR RESPONSE 1) was up-regulated; six orthologs of early auxin-responsive *AUXIN/INDOLEACETIC ACID-INDUCED PROTEINs* (*Aux/IAAs)* were up-regulated including *IAA2, IAA7, IAA9, IAA12, IAA16* and *IAA28* while three *IAA17* and one *IAA15* were down-regulated. According to the auxin pathway description in the KEGG database, *Aux/IAAs* are regulated at multiple levels, consistent with their critical role in maintaining proper auxin response (Figure S3). In addition to the *Aux/IAAs, three* orthologs of *AUXIN RESPONSE FACTORs (ARFs)* were up-regulated including *ARF6, ARF10* and *ARF16*. Except for one up-regulated ortholog of SMALL AUXIN UP-RNA 20 (*SAUR20*), 15 orthologs of *SAURs* were down-regulated including one *SAUR19, SAUR23, SAUR24, SAUR36, SAUR61, SAUR62*, two *SAUR66*, four *SAUR20s*, and three *SAUR21s* (Figure S3). In addition, significant differences in expression of *NIT2, TSB1, TSB2*, and *YUCCA* genes that are involved in regulating the IAA biosynthetic pathway were detected in the DB_S to Sx_S comparison (Table S4).

Finally, significant expression differences were found for the ABA related genes *PYL*/*PYR, PP2C, SRK2*, and *ABF* between DB and SX (Table S5) suggesting some involvement of ABA signaling.

#### Validation of RNA-Seq data by quantitative RT-PCR (qRT-PCR)

**Figure 6:**
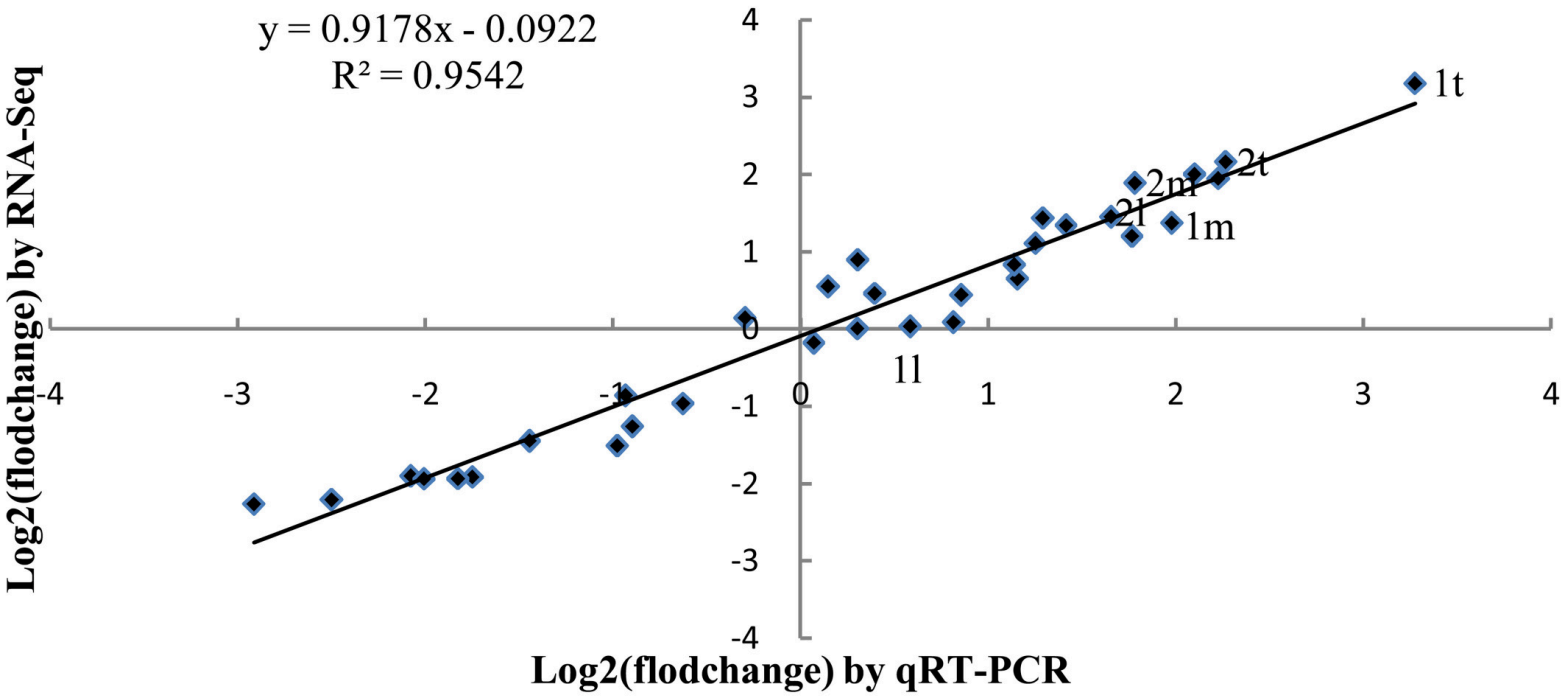
Comparison of results obtained via RNA-Seq and qRT-PCR for 10 genes expressed at different locations along the leaf sampled from overlapping and outward-curling morphotypes. 1t, 1m, and 1l: *BrGH3.12* expression at top, middle, and basal position, respectively; 2t, 2m, and 2l: *BrABF1* expression at top, middle, and basal position, respectively.

To verify the RNA-Seq data, we performed qRT-PCR on 10 unigenes at three leaf positions in overlapping and outward-curling plant types. In addition to *BrGH3.12* and *BrABF1*, eight unigenes were randomly selected. As shown in Figure 6, all 10 genes displayed the same expression patterns in the qRT-PCR assays as in the RNA-Seq analysis. The Pearson correlation coefficient between the two methods was 0.9542, confirming the quality of the RNA-seq data.

### Concentrations of IAA and ABA

**Figure 7:**
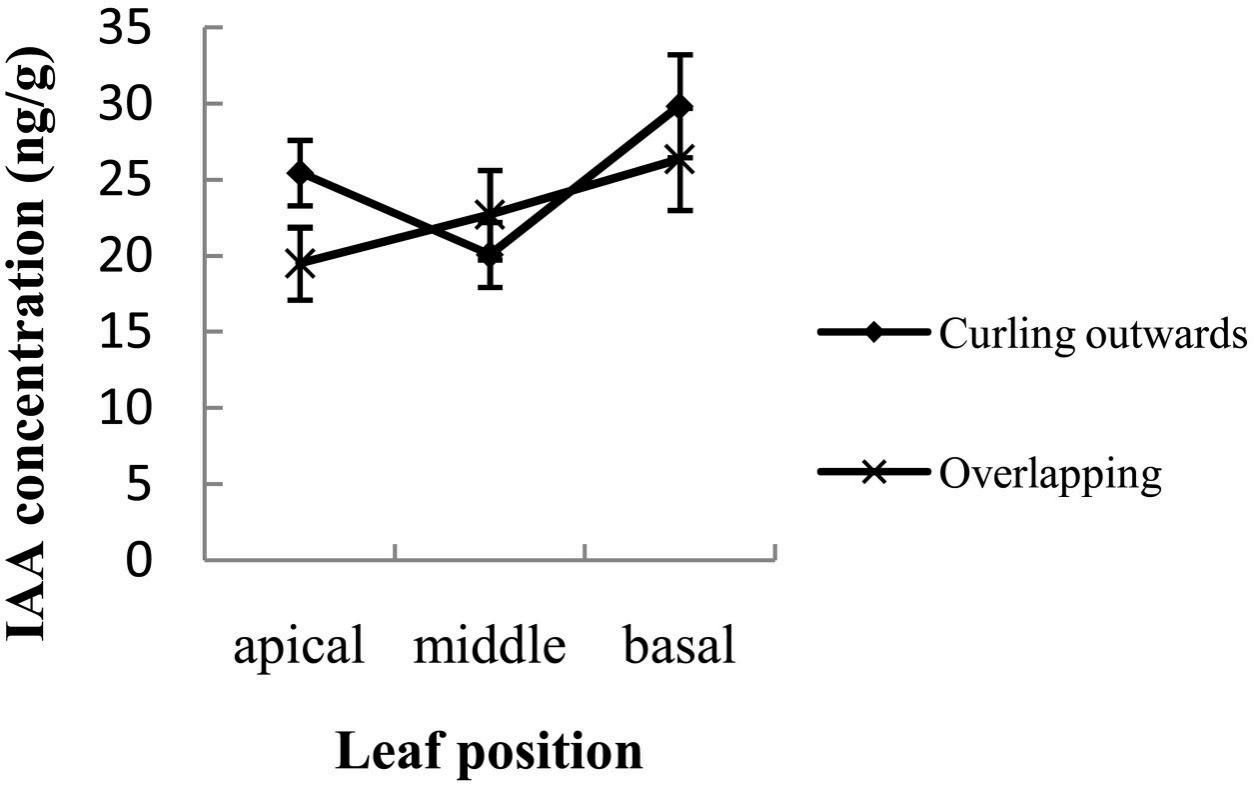
Concentrations of IAA measured at 3 positions within the leaf sampled from plants showing overlapping or outward-curling morphotypes of head formation.

At the early heading stage, the IAA concentration was significantly higher for the outward-curling type than for the overlapping type when measured at the apical region of the leaf (*P* = 0.03). In contrast, the differences in IAA levels were not significant between morphotypes when measured at the middle (*P* = 0.28) and basal (P = 0.27) segments of the leaf (Figure 7). This trend in IAA concentrations across leaf positions and between overlapping and outward-curling types is consistent with the variation in expression of *BrGH3.12* detected under the same parameters. In the outward-curling type, IAA concentrations were 1.27-fold higher in the apical region than in the middle while the basal portion contained less IAA than the apical region (Figure 7). However, in the overlapping type, the IAA concentration was 0.86-fold lower at the apical than in the middle region and greater at the basal region compared to the apical region (Figure 7).

**Figure 8:**
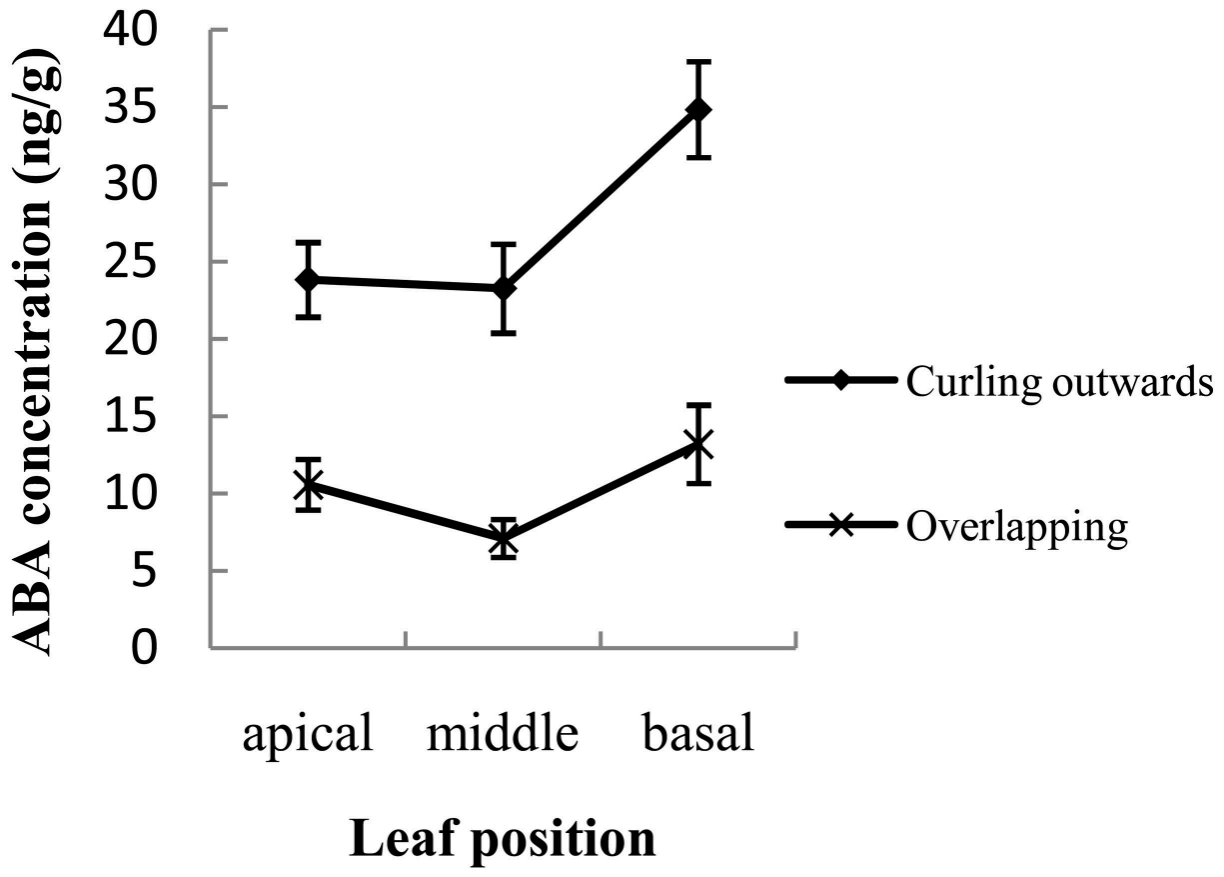
Concentrations of ABA measured at 3 positions within the leaf sampled from plants showing overlapping or outward-curling morphotypes of head formation.

At each corresponding position, the ABA concentration was always significantly higher in the outward-curling type than in the overlapping type (*P* < 0.01) (Figure 8). When comparisons were made at each leaf position, the apical also had consistently more ABA than either the middle or basal portions, regardless of morphotype (Figure 8). Within an individual type, the ABA concentration at the apical region of the leaf was 1.01-fold higher than the middle for the outward-curling type and 1.52-fold higher than the middle for the overlapping type (Figure 8). In the latter type, the apical region of the leaf tended to grow more slowly than the middle portion; this discrepancy was supported by the difference in levels of IAA and ABA measured at those positions. Based on the Gaussian curvature theory, leaves for which marginal regions grow slower than the central regions form a cup-like shape with positive Gaussian curvature (Nath et al., 2003). Therefore, one would expect to see this difference in growth rates manifested by leaves that curl inward and overlap when the head is forming. By comparison, faster growth at the apical region of the leaf rather than in the middle would naturally lead to an outward-curling pattern.

## Discussion

The heading type is an important agricultural trait in Chinese cabbage. To investigate the mechanism of overlapping and outward curling heading type, the self-crossed progeny of one natural microspore-derived doubled haploid plant were examined. The candidate biological pathway contributing to this trait was identified by Seq-BSA and RNA-Seq analysis, highlighting the success of this joint approach. Furthermore, the concentrations of related metabolites in the pathway were detected. The results from the genomic, transcript and metabolite level analyses support a role for auxin and ABA signaling during heading type specification.

Merging divergent genomes into a single nucleus can trigger a “highly-programmed sequence of events within the cell that serves to cushion the effect of [genomic] shock” (McClintock, 1984). Genetic alterations have been reported for some synthetic polyploids, such as *Arabidopsis thaliana* (Comai et al., 2000; Madlung et al., 2002), *Triticeae* (Ozkan et al., 2001; Han et al., 2003; Ma et al., 2004), and *Brassica* (Song et al., 1995). The introgression of alien chromosome fragments can also severely affect the accepting genome, thereby triggering genetic and genomic changes (Liu et al., 2015). In this study, heading type showed segregation in self-crossed progenies from a single natural doubled haploid plant that integrated cabbage chromosome segments in the Chinese cabbage genome background. Similar segregations in self-crossed progenies have been derived from other Chinese cabbage introgression lines that integrated various segments from different chromosomes of cabbage (Kang et al., 2015; Li, 2015; Yan et al., 2015; Geng et al., 2016). These findings provide further evidence that trait segregation is caused by variations in receptor genomes that are induced by genomic shock and not, in the case of Chinese cabbage, by some genes added from a donor cabbage. In addition, using the Seq-BSA data, we mapped the remaining reads to the cabbage reference genome after mapping reads to the Chinese cabbage reference genome but did not obtain a candidate region. This led us to focus on the accepting Chinese cabbage genome in this study.

Auxin influences many processes in the plant including cell division, elongation, and differentiation and is active throughout the development of leaves (Ludwig-Muller, 2011; Garrett et al., 2012), flowers (Cheng et al., 2006; Tabata et al., 2009), fruits (Eklund et al., 2010; Shalom et al., 2014), and roots (Overvoorde et al., 2010; Jones and Ljung, 2012). Auxin also plays an important role during embryogenesis (Cheng et al., 2007; Braun et al., 2008; Wang, W. et al., 2011), hypocotyl elongation (Lilley et al., 2012), shoot regeneration (Qiao et al., 2012), branch point density (Esteve-Bruna et al., 2013), and plant responses involving phototropism, gravitropism, and apical dominance (Horiguchi et al., 2006; Cho et al., 2007).

During leaf development, auxin affects vein formation (Scarpella et al., 2006; Li et al., 2008), the number of palisade mesophyll cells, and air spaces within the spongy mesophyll (Esteve-Bruna et al., 2013). Auxin modulates the balance between adaxial and abaxial cell growth, giving rise to curly phenotypes (Bowman et al., 2002; Byrne, 2005; Qin et al., 2005; Izhaki and Bowman, 2007; Kidner and Timmermans, 2007; Wu et al., 2007, Braun et al., 2008; Wu et al., 2008; Husbands et al., 2009; Liu et al., 2011), epinastic phenotypes (Qin et al., 2005), and variations in leaf angle (Song et al., 2009; Bian et al., 2012). Cheng et al. (2016) reported that auxin-related genes are strongly associated with the leaf-curling trait (heading or non-heading) in *Brassica rapa*. Our data suggest that the significant differences in IAA concentrations and venation patterns between morphotypes at the apical region of the leaf is largely responsible for the determination of outward-curling vs. overlapping types of head formation. This is also supported by the Seq-BSA and RNA-Seq analyses that identified genes involved in the auxin pathway.

As an early-auxin-response gene, *GH3* encodes indole-3-acetic acid-amido synthetase and functions in maintaining auxin homeostasis by conjugating excess IAA to various amino acids (Hagen and Guilfoyle, 1985; Staswick et al., 2002). The *OsGH3* gene family member in rice (*Oryza sativa*) has a crucial role in controlling leaf inclination (Zhang, L. Y. et al., 2009; Zhang, S. W. et al., 2009; Du et al., 2012; Zhao, S. Q. et al., 2013), while an auxin-response factor, *OsARF19*, modulates rice leaf angle through positive regulation of *OsGH3-5* and *OsBRI1* (Zhang et al., 2015). In *Brassica rapa, ARF3* is also associated with heading and non-heading (Cheng et al., 2016). Overexpression of *OsGH3* family genes in rice results in a decrease in free-IAA and similar morphological phenotypes that include dwarfism, greater leaf angle, shorter leaves, smaller panicles, and fewer crown roots and root hairs (Ding et al., 2008; Domingo et al., 2009; Zhang, S. W. et al., 2009; Fu et al., 2011; Du et al., 2012; Zhao, S. Q. et al., 2013). Based on the results from the combined Seq-BSA and RNA-Seq analysis, we selected *BrGH3.12* as a candidate gene. *BrGH3.12* expression at the apical region of the leaf was significantly higher in the overlapping type than in the outward-curling type, a trend that was opposite to the levels of IAA. However, neither the expression of *BrGH3.12* nor IAA concentrations differed between morphotypes when we examined the middle and basal portions of the leaf. The relationship between *BrGH3.12* expression levels and IAA concentrations are consistent with previous studies.

The phytohormone ABA serves as an endogenous messenger that plays a key role in plant responses to environmental stress and several developmental processes, including root growth (McAdam et al., 2016), seed maturation, and dormancy (Yoshida et al., 2006; Miyakawa et al., 2013). Endogenous ABA has an obvious and ubiquitous inhibitory effect on shoot growth, which is thought to stem primarily from its ability to induce stomatal closure and, ultimately, decrease assimilation (Tardieu et al., 2010). The balance of IAA and ABA homeostasis plays a crucial role in plant development and diverse stress responses in rice (Du et al., 2012). In our study, the concentrations of auxin and ABA were significantly different between morphotypes. Therefore, both *BrGH3.12*, an early auxin-responsive gene, and *BrABF1*, a gene involved in ABA signaling, appear to be suitable candidates for regulating heading type in Chinese cabbage.

## Ethics statement

The study was approved by Hebei Agricultural University, China. All provided written informed consent.

## Author contributions

AG and SS conceived the original screening and research plans; AG and JZ supervised the experiments; AG, CM, HD, and YC performed most of the experiments; XC and YL provided technical assistance; AG, YW and LW designed the experiments and analyzed the data; AG conceived the project and wrote the article with contributions from all authors; JZ and SS supervised and reviewed the writing.

### Conflict of interest statement

The authors declare that the research was conducted in the absence of any commercial or financial relationships that could be construed as a potential conflict of interest.

## Supplementary material

Figure S1 Leaf growth over time for plants showing overlapping or outward-curling morphotype.

Figure S2 Sampling segments for leaves analyzed via RNA-seq.

Figure S3 Difference genes expression in the auxin pathway.
