# Supplementary figures and images for: Coupling Seq-BSA and RNA-Seq Analyses Reveal the Molecular Pathway and Genes Associated with Heading Type in Chinese Cabbage
Source: Front Genet. 2017 Dec 12;8:176. doi: 10.3389/fgene.2017.00176 (PMC5733010; doi:10.3389/fgene.2017.00176)

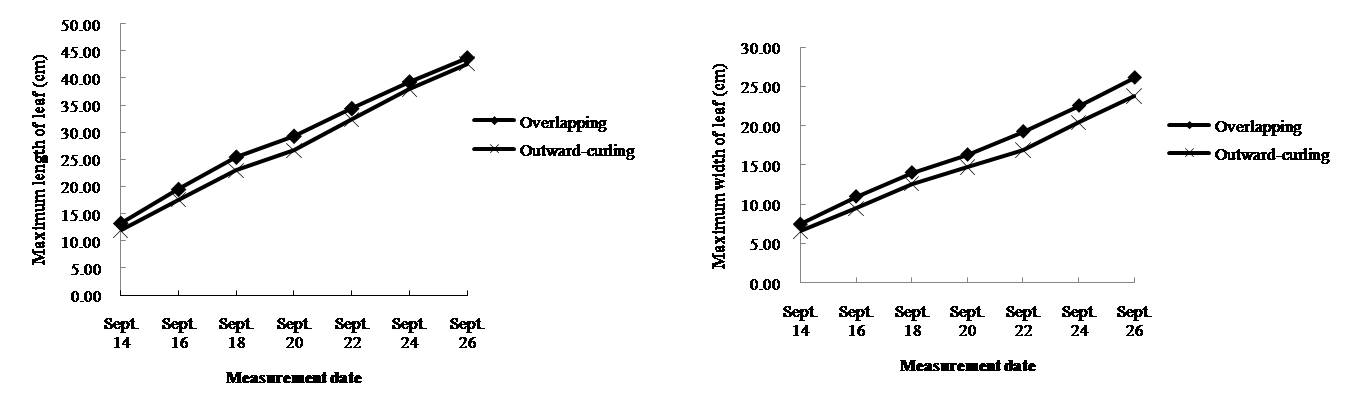

Supplement: Figure S1 — Leaf growth over time for plants showing overlapping or outward-curling morphotype. [file Image1.jpeg]

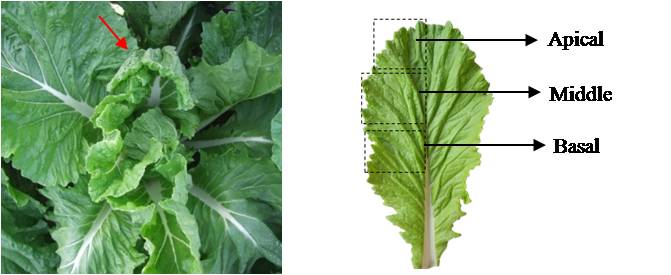

Supplement: Figure S2 — Sampling segments for leaves analyzed via RNA-seq. [file Image2.jpeg]

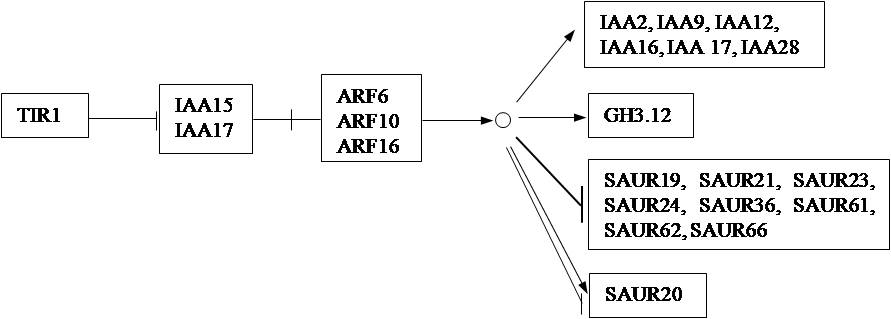

Supplement: Figure S3 — Difference genes expression in the auxin pathway. [file Image3.jpeg]
