# Supplementary material for: Coupling Seq-BSA and RNA-Seq Analyses Reveal the Molecular Pathway and Genes Associated with Heading Type in Chinese Cabbage
Source: Front Genet. 2017 Dec 12;8:176. doi: 10.3389/fgene.2017.00176 (PMC5733010; doi:10.3389/fgene.2017.00176)
Supplement: Supplementary file 4 [file Table1.DOCX]

| Gene ID | Primer sequence(3'-5') | Primer sequence(5'-3') | Amplification fragment size (bp) |
| --- | --- | --- | --- |
| Bra018750 | TTTACGCTTCTTCCGAGTCAA | GTCCACAACATCACCATTGC | 140 |
| Bra018800 | GAATCAAGCACCAGCAACAA | CTCCTCTTCCCATAGCAGCA | 152 |
| Bra018722 | CCACAAGAACCAGTCCTC | GCCACTATCAACCACCAA | 162 |
| Bra039362 | GCAGAGGCAGTTCTTGAA | ACCTTGTTCTCCTTCATCAG | 104 |
| Bra023317 | TCAGCACAGCAAGCAAGA | GCGTTAGAACCACCTCCT | 201 |
| Bra039652 | TTCACTAACGGCGACATG | GGCTCTACGGCAATAAGG | 111 |
| Bra004420 | TGTTGTCGTTGCCCTTGTTA | GTCCATTCTGGGAGTCGGTA | 140 |
| Bra040495 | TGCGTCCTAAGTTTGCTCCT | TGATTGGTTGCTGTTGAAGC | 110 |
| Bra018844 | TATACAGGATGGCATTGACT | ACAGGAATTAGCACTTAGGT | 181 |
| Bra018813 | TTTGTTGGTGGACTTGATGC | TTTGTTGGTGGACTTGATGC | 142 |
| Actin | TGTAGCACCTGAAGAGCA | AGAACAGCCTGAATAGCG | 119 |

Table S1 Primers used in qRT-PCR
